# Rheological Properties and Structural Stability of Sodium Caseinate-Based Inks Reinforced with Pectin and κ-Carrageenan for 6-Axis Robotic 3D Printing

**DOI:** 10.3390/foods15203709

**Published:** 2026-10-18

**Authors:** Emre Vay, Veli Ozbolat

**Affiliations:** 1 Department of Mechanical Engineering, Ceyhan Engineering Faculty, Cukurova University, 01950 Adana, Türkiye; emrevy@hotmail.com; 2 Department of Tissue Engineering, Institute of Natural and Applied Sciences, Cukurova University, 01250 Adana, Türkiye; 3 Biotechnology Research and Application Center, Cukurova University, 01250 Adana, Türkiye

**Keywords:** sodium caseinate, pectin, κ-carrageenan, 3D food printing, six-axis robotic printing, food texture, rheology, shape fidelity

## Abstract

Formulating printable and structurally stable edible inks is a challenge in 3D food printing. This study investigated the effects of combined pectin and κ-carrageenan concentrations on skim milk and sodium caseinate-based inks. Three formulations with varying polysaccharide complex (PC) concentrations (Low-PC, Med-PC, and High-PC) were characterized using Fourier-transform infrared spectroscopy (FTIR), scanning electron microscopy (SEM), rheological tests, and texture profile analysis (TPA) followed by six-axis robotic printing. Higher PC concentrations produced more compact freeze-dried matrices and increased rheological strength and textural hardness. FTIR spectral changes suggested possible non-covalent interactions. All formulations exhibited shear-thinning behavior, while High-PC showed the highest mean structural recovery, although the difference between High-PC and Med-PC was not statistically significant (*p* > 0.05). During printing of a star geometry, Low-PC spread beyond the target area, whereas High-PC produced smaller structures. Med-PC achieved the closest agreement with the target area (96.16% area-based shape fidelity). After 2 h of immersion, swelling ratios were 267.81%, 313.81%, and 285.12% for Low-PC, Med-PC, and High-PC, respectively, without statistically significant differences (*p* > 0.05). After 5 h, High-PC retained a star-like structure, whereas Low-PC and Med-PC largely lost their printed geometries. These findings highlight a trade-off between printing accuracy and post-printing structural stability.

## 1. Introduction

Additive manufacturing, widely known as three-dimensional (3D) printing, has gained significant attention in recent years due to its suitability for personalized nutrition and its ability to reproducibly fabricate complex geometric designs that are difficult to achieve using traditional food processing methods [1,2,3]. Although extrusion-based printing is the most widely used method in 3D food printing due to its processing versatility [4,5], conventional Cartesian systems are largely restricted to planar toolpaths and fixed deposition orientations, hindering the creation of self-supporting complex structures [6]. Integrating multi-axis robotic arms with extra degrees of freedom provides greater flexibility in controlling the deposition path, enabling the fabrication of intricate geometries and structures that are challenging to produce using conventional Cartesian printing systems [7,8]. This directional flexibility is particularly important for food printing, as the extruded material must maintain a stable filament and geometry under changing deposition directions. This means the mechanical advantages of multi-axis robotic printing cannot be considered independently of the flow and structural properties of the printing ink.

Despite the advantages of extrusion-based multi-axis robotic printing systems, the successful fabrication of food structures heavily depends on the rheological properties of the printing material [9]. For successful printing, the material must flow smoothly through the nozzle under applied pressure while retaining its shape immediately after deposition [10]. Exhibiting shear-thinning behavior—where apparent viscosity decreases with increasing shear rate—facilitates material flow through the nozzle under the high-shear conditions generated during extrusion while reducing the required extrusion pressure. At the same time, sufficient viscoelasticity and yield stress help the material retain its shape after deposition and support subsequent layers without collapsing. Furthermore, the rebuilding of the internal network upon the removal of shear stress, referred to as structural recovery, is essential for preserving the intended form immediately after printing. Therefore, the rheological properties of food inks are among the most critical factors determining extrudability and shape fidelity in extrusion-based 3D food printing [4,5,9,11,12]. These requirements create a fundamental balance between extrusion flowability and post-deposition structural stability, which becomes particularly important when complex toolpaths and deposition orientations are used in multi-axis robotic printing. As a result, evaluating rheological properties together with the actual printed geometry is necessary to determine whether a food formulation is suitable for robotic extrusion printing.

Among food-grade biopolymers, milk proteins and polysaccharides have attracted growing interest in 3D food printing due to their nutritional value and functional properties [11]. Caseins, which constitute a major fraction of milk proteins, exhibit functional properties such as water binding, emulsification, and gel formation owing to their amphiphilic nature [13,14]. Sodium caseinate, in particular, is a highly soluble protein with favorable functional properties, making it a suitable ingredient for personalized nutrition and structured food systems. However, despite these advantages, formulations composed solely of milk and sodium caseinate may not provide sufficient rheological properties for reliable extrusion-based 3D printing [15,16]. To overcome these rheological limitations and achieve an appropriate balance of flowability and post-deposition stability required for extrusion-based 3D printing, additional structuring agents are required in the formulation. The addition of polysaccharides improves the rheological and structural properties of protein-based dairy formulations via changes in viscosity, water immobilization, and protein–polysaccharide interactions, resulting in potentially suitable printable composite food inks [17,18,19]. However, increasing the polysaccharide concentration improves structural resistance but also increases extrusion resistance. Therefore, developing printable protein–polysaccharide inks requires an optimal balance rather than simply maximizing viscosity or gel strength.

High-methoxyl pectin and κ-carrageenan are food-grade polysaccharides frequently used to modify the rheological and structural properties of protein-based food systems [20,21]. High-methoxyl pectin acts as a thickening and stabilizing agent that primarily enhances the viscosity and water retention of the formulation while also contributing to the shear-thinning behavior [20,22,23]. On the other hand, κ-carrageenan acts as a robust gelling agent widely used for its high water-binding capacity and its ability to form rigid three-dimensional networks [21,24]. As a result, adding high-methoxyl pectin and κ-carrageenan to milk protein formulations adjusts the rheological and mechanical properties of the food ink [25]. However, simply increasing the polysaccharide concentration does not always mean better printability. Excessive amounts increase extrusion resistance, while insufficient levels cause the printed shape to spread or collapse [5]. Therefore, balancing these agents is essential to achieve smooth extrusion and high shape fidelity in 3D food printing.

High-methoxyl pectin and κ-carrageenan are therefore considered together in this study as a polysaccharide complex (PC) to investigate the effect of increasing PC concentration, rather than isolating their individual roles. Although protein–polysaccharide systems have been studied in extrusion-based printing, the relations among polysaccharide concentration, structural characteristics at different scales, and the dimensional fidelity of complex structures produced by multi-axis robotic printing remain poorly understood.

Characterization at multiple structural levels is required to understand the relationship between formulation composition and printing performance. Fourier-transform infrared spectroscopy (FTIR) provides information about the functional groups and molecular interactions associated with protein and polysaccharide components [26,27]. In addition, changes in the microstructure and the resulting networks can be observed using scanning electron microscopy (SEM). These structural features are closely linked to the macroscopic mechanical properties of the material. Since the mechanical properties of the material can affect its processing and oral textural characteristics in food applications, defining parameters such as hardness, springiness, cohesiveness, gumminess, chewiness, and resilience is important. Therefore, texture profile analysis (TPA), which captures the material’s mechanical response under deformation, effectively complements both rheological and microscopic characterizations [28,29,30].

Although rheological measurements of flow behavior, viscoelastic properties, and structural recovery provide insights into the printability of food inks, actual printing success must be evaluated based on the produced geometry. In this context, it is important to quantitatively determine shape fidelity, which indicates how closely the printed geometry matches the target design [31]. Water removal during drying can significantly alter the structure and mechanical properties of biopolymer networks, while water uptake can cause disintegration and dimensional changes. Therefore, for food applications, evaluating the drying and aqueous stability of the structure—in addition to its initial shape fidelity—provides further information about the post-printing structural stability of the printed food. However, studies that combine molecular and microstructural characterization with rheology, texture, structural recovery, multi-axis robotic extrusion, quantitative shape fidelity, and post-printing stability within a single protein–polysaccharide food ink system remain limited.

Therefore, the main scientific question of this study is whether increasing the concentration of a high-methoxyl pectin–κ-carrageenan complex (PC) can optimize the balance between extrusion behavior, post-deposition structural stability, and dimensional fidelity in six-axis robotic printing. To address this question, three PC concentrations were investigated in a sodium caseinate-based formulation. A comprehensive characterization approach was used to examine the relationship between formulation properties and actual printing performance. The potential molecular interactions and microstructures of the composite inks were investigated using FTIR and SEM, respectively. The flow behaviors, viscoelastic properties, and structural recovery of the inks were assessed through steady-shear, amplitude sweep, frequency sweep, and three-interval thixotropy tests to simulate the extrusion and post-extrusion phases. Additionally, TPA was performed to examine the macroscopic mechanical properties of the formulations and to characterize the instrumental textural properties relevant to food texture. To evaluate the extrudability and quantitatively determine the shape fidelity of the formulations, a three-dimensional five-pointed star with a single apex was printed. This complex geometry was specifically chosen to challenge the deposition capability of the multi-axis robotic arm and evaluate its ability to fabricate complex geometries. Shape fidelity was calculated based on the dimensional accuracy of the printed geometry relative to the target design in terms of length and area. Finally, the post-printing structural integrity of the produced structures was investigated by measuring their weight loss during drying, evaluating their swelling behavior, and visually assessing their morphological behavior during immersion. By integrating these measurements, this study aims to clarify how increasing PC concentration modifies the structural characteristics of sodium caseinate-based food inks and how these changes affect rheological behavior, robotic extrusion, dimensional fidelity, and post-printing structural stability. These findings are expected to provide formulation-level guidance for the development of protein–polysaccharide food inks for multi-axis robotic 3D printing.

## 2. Materials and Methods

### 2.1. Materials

Skim milk powder (35% protein, 52% carbohydrates, and 1% fat) and sodium caseinate (86.5% protein, 2% fat, and 0.3% lactose, with a pH range of 6.6–7.0) were purchased from Alfasol Food Technologies (Istanbul, Turkiye). Apple-derived high-methoxyl pectin (E440, France origin, Alfasol, Istanbul, Turkiye) and refined κ-carrageenan (E407, South Korea origin, Benosen, Istanbul, Turkiye) were used as a rheological modifier and a gelling agent, respectively. All ingredients were of food-grade quality and used without further purification. Distilled water was used for the preparation of all hydrogel formulations.

### 2.2. Preparation of the Inks

While the sodium caseinate concentration (8.00 wt%) was kept constant, three formulation groups were prepared by varying the concentration of the polysaccharide complex (PC), which consists of high-methoxyl pectin and κ-carrageenan: Low-PC (2.00 wt% total; 1.00 wt% pectin and 1.00 wt% κ-carrageenan), Med-PC (3.25 wt% total; 2.00 wt% pectin and 1.25 wt% κ-carrageenan), and High-PC (4.50 wt% total; 3.00 wt% pectin and 1.50 wt% κ-carrageenan). The amounts of skim milk powder and distilled water were calculated based on the remaining mass after accounting for sodium caseinate and PC. This remaining portion was divided between skim milk powder and distilled water at a mass ratio of 1:9, following the manufacturer’s recommended reconstitution ratio for skim milk powder. Thus, the total formulation mass was maintained at 100 wt% for all groups. The resulting compositions are presented in Table 1.

The dry ingredients (skim milk powder, sodium caseinate, high-methoxyl pectin, and κ-carrageenan) were thoroughly pre-blended to prevent agglomeration during hydration. Distilled water was pre-heated to 75 °C, and the powder blend was gradually added into the vortex and initially stirred at 70 °C at 500 rpm for 30 min to achieve complete solubilization [32,33]. Additional manual mixing using a spatula was performed for all formulations to facilitate uniform dispersion and minimize agglomeration. After mixing, the prepared inks were placed in a refrigerator for 40 min to accelerate cooling and facilitate the dissipation of entrapped air bubbles. During this period, the sample temperature decreased to approximately 20 °C; the samples were not maintained at 4 °C. The samples were then allowed to rest at room temperature for about 30 min before being transferred to syringes for 3D printing or used for characterization.

To evaluate changes in pH during preparation and cooling, the pH of the inks was measured using a SevenCompact pH meter (Mettler Toledo, Greifensee, Switzerland). Measurements were performed at three stages: immediately after preparation, immediately after removal from the refrigerator, and after standing at room temperature for approximately 30 min.

### 2.3. Chemical Characterization

The functional groups and possible non-covalent interactions among the components were investigated using an FT/IR-6700 FTIR spectrometer equipped with an attenuated total reflectance (ATR) accessory (Jasco, Tokyo, Japan). The pure raw materials (skim milk powder, sodium caseinate, high-methoxyl pectin, and κ-carrageenan) were directly analyzed in their native powder form to establish baseline reference spectra. The hydrogel samples were lyophilized prior to analysis. Spectra were recorded in the wavenumber range of 4000 to 500 cm^−1^ with a resolution of 4 cm^−1^, accumulating 32 scans per sample at room temperature.

### 2.4. Microstructural Characterization

The microstructural morphology of the formulations was examined using a Quanta 650 Field Emission Scanning Electron Microscope (FEI Company, Hillsboro, OR, USA). The prepared hydrogels (Low-PC, Med-PC, and High-PC) were first pre-frozen at −25 °C overnight, followed by lyophilization (−50 °C, 48 h) to minimize structural changes during drying. The dried specimens were carefully fractured to expose their internal cross-sections and sputter-coated with a thin gold–palladium layer to enhance surface conductivity and prevent electron charging artifacts. Micrographs were captured at an accelerating voltage of 15 kV with magnifications ranging from 500× to 10,000× to observe the protein–polysaccharide interactions and pore architecture.

### 2.5. Rheological Characterization

Rheological properties of the ink formulations were determined using an MCR 302e rheometer (Anton Paar, Graz, Austria) equipped with a 25 mm parallel-plate geometry at a fixed gap of 1 mm. All measurements, except the temperature sweep tests, were conducted at 25 °C. A Peltier-controlled system (H-PTD220) with an active hood was used to control the measurement temperature and minimize temperature gradients. All data acquisition and analysis procedures were performed using RheoCompass software (Anton Paar, Austria). Measurements were performed in triplicate for each formulation, and the results were expressed as mean ± standard deviation.

#### 2.5.1. Steady Shear Flow Test

The flow behavior of the ink formulations was evaluated using steady shear measurements over a shear rate range of 0.1–1000 s^−1^. However, to eliminate measurement noise and experimental artifacts typically observed at extreme boundary conditions, the initial and final data points were excluded. Therefore, the apparent viscosity curves were only plotted for the reliable shear rate range of 0.185 to 541 s^−1^. To determine the shear-thinning characteristics of the formulations, the shear stress–shear rate data within this range were fitted to the Power-Law model (Equation (1)):

(1) $$\tau =K\cdot \;{\dot{\gamma }}^{n}$$

where τ is the shear stress (Pa), $\dot{\gamma }$ is the shear rate (s^−1^), K is the consistency index $\left(Pa\cdot s^{n}\right)$, and *n* is the dimensionless flow behavior index.

Additionally, the Herschel–Bulkley model (Equation (2)) was evaluated to compare its fitting performance with that of the Power-Law model:

(2) $$\tau =\tau_{0}+K\cdot \;{\dot{\gamma }}^{n}$$

where $\tau_{0}$ is the fitted yield stress (Pa) [34]. The Herschel–Bulkley model was fitted using nonlinear regression, with $\tau_{0}$ constrained to non-negative values ($\tau_{0}\geq 0$). Model fitting performance was evaluated using the coefficient of determination ($R^{2}$).

#### 2.5.2. Dynamic Oscillatory Tests

Dynamic oscillatory tests were performed to evaluate the structural stability and viscoelastic properties of the inks. The yield stress, the flow stress, and the linear viscoelastic region (LVR) were determined through an amplitude sweep test in a strain range of 0.01–100% at a fixed angular frequency of 10 rad/s. The LVR was defined as the strain where the storage modulus (*G*′) deviated by 3% from its initial plateau. The yield stress was determined from the amplitude sweep data using the onset point method on the logarithmic *G*′ curve in the rheometer software. The flow stress was defined as the stress at the crossover point of *G*′ and the loss modulus (*G*″) (*tan δ* = 1). At a constant strain selected within the determined LVR for each formulation, a frequency sweep between 100 and 1 rad/s was performed to analyze *G*′ and *G*″, as well as the loss factor (*tan δ*). The *G*′ and *tan δ* values at 10 rad/s were used for quantitative comparison among formulations.

#### 2.5.3. Three-Interval Thixotropy Test (3ITT)

A 3ITT was performed to evaluate the structural breakdown and recovery behavior of the ink formulations under deformation conditions intended to represent the extrusion-based 3D printing process. During the first interval, a low oscillatory strain of 0.5% was applied at an angular frequency of 10 rad/s for 120 s to characterize the initial structure. In the second interval, the strain was increased to 100% for 120 s at the same angular frequency to induce structural disruption. Subsequently, the strain was reduced to 0.5% for 300 s in the third interval to monitor structural recovery.

The structural recovery percentage was calculated using Equation (3):

(3) $$Recovery\;\left(\%\right)=\frac{G_{III,t}^{\prime }}{G_{I,120}^{\prime }}\times 100,$$

where $G_{III,t}^{\prime }$ represents the storage modulus measured at 60 or 300 s after the beginning of the third interval, and $G_{I,120}^{\prime }$ represents the storage modulus recorded at 120 s, corresponding to the final measurement point of the first interval.

#### 2.5.4. Temperature Sweep Test

A temperature sweep test was conducted to evaluate the temperature-dependent viscoelastic behavior of the inks and determine whether warming was associated with a distinct change in their structure. The storage modulus (*G*′) and loss modulus (*G*″) were recorded as the temperature was increased from 4 °C to 30 °C at a constant heating rate of 2 °C/min, applying a constant oscillatory strain within the LVR and a fixed angular frequency of 10 rad/s. Although the ink temperature decreased to approximately 20 °C during the 40-min refrigeration step, the temperature sweep was extended to a starting temperature of 4 °C to examine the viscoelastic response over a broader temperature range, including the lower temperatures associated with refrigeration. The results were used to assess changes in the relative contributions of elastic and viscous behavior during heating.

### 2.6. Mechanical and Textural Characterization

The textural properties of the formulated hydrogels were evaluated using a TA.XT Plus Texture Analyzer (Stable Micro Systems, Godalming, UK) equipped with a 5 kg load cell and a 36-mm diameter stainless steel cylindrical probe. A standard double-compression TPA was performed. The trigger force was set to 5 g to ensure accurate surface detection. Immediately after preparation, the inks were transferred into plastic syringes, which served as molds for preparing cylindrical specimens. The filled syringes were placed in a refrigerator for 40 min, during which the ink temperature decreased to approximately 20 °C, and were subsequently kept at room temperature for about 30 min. The inks were then carefully extruded from the syringes and cut into cylindrical specimens measuring 15 mm in height and 17.5 mm in diameter. These specimens were then subjected to a 40% compressive strain with a 5-s resting period between the two consecutive compression cycles. The pre-test, test, and post-test speeds were maintained at 1.0, 1.2, and 2.0 mm/s, respectively. All measurements were performed in triplicate at room temperature (25 ± 1 °C), and data acquisition was carried out using Exponent software (Stable Micro Systems, UK). Key textural parameters such as hardness, springiness, cohesiveness, gumminess, chewiness, and resilience were calculated from the force–time curves. These measurements were performed on syringe-molded specimens rather than 3D-printed constructs and therefore represent the bulk textural properties of the formulations, not the inter-filament or interlayer bonding of printed structures.

### 2.7. Six-Axis Robotic 3D Printing and Shape Fidelity Evaluation

**Figure 1:**
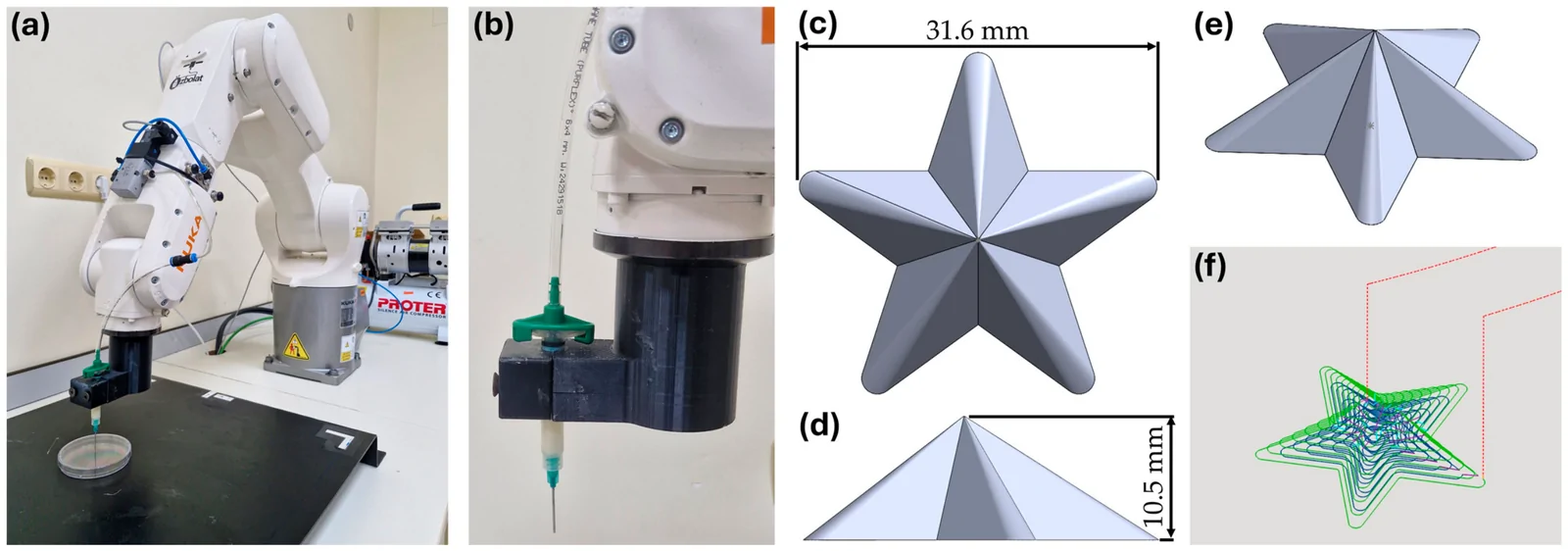
Overview of the robotic 3D food printing system and geometrical design of the star-shaped construct. (**a**) General view of the 6-axis robotic arm configured for extrusion-based printing. (**b**) Close-up view of the extrusion mechanism. (**c**) Top and (**d**) side views of the computer-aided design (CAD) model detailing lateral and vertical dimensions, respectively. (**e**) Isometric 3D view of the target geometry. (**f**) Corresponding toolpath generated for the layer-by-layer extrusion process.

A 6-axis KR 6 R900-2 robotic arm (Kuka, Augsburg, Germany) modified with a custom pneumatic extruder was used for the 3D printing experiments (Figure 1a). The prepared inks were loaded into 3 mL syringes fitted with 18 G nozzles (inner diameter: 0.84 mm) (Figure 1b). All printing steps were performed at ambient temperature (25 ± 1 °C) and a constant travel speed of 3.75 mm/s. Because the formulations exhibited different flow resistances, preliminary printing trials were conducted to adjust the extrusion pressure for each formulation while targeting approximately comparable material output rates. Accordingly, extrusion pressures of 55, 160, and 310 kPa were used for Low-PC, Med-PC, and High-PC, respectively. The approximate mass extrusion rates measured under these conditions were 0.002905 ± 0.000062, 0.002972 ± 0.000102, and 0.003076 ± 0.000116 g/s for Low-PC, Med-PC, and High-PC, respectively. These formulation-specific pressures were selected to achieve approximately comparable mass extrusion rates at a fixed printing speed, accounting for differences in the flow resistance of the formulations. The mass extrusion rate was calculated by dividing the total mass of extruded material by the printing program duration.

To demonstrate the overall printability and evaluate the shape fidelity of the hydrogel inks, a five-pointed star geometry was chosen as the target construct. The computer-aided design (CAD) model was created using SOLIDWORKS (version 2025 SP0.0) (Dassault Systèmes, Vélizy-Villacoublay, France) (Figure 1c–e), while the toolpaths for the robotic motion were generated using SprutCAM X Robot (version 17.0.21) (SprutCAM Tech., Limassol, Cyprus) (Figure 1f). The target dimensions of the star were 31.6 mm in overall width and 10.5 mm in height. A continuous solid toolpath strategy with an 80% stepover was employed to produce a continuous infill pattern. The total printing time to complete a single sample was approximately 6 min.

Following the 3D printing process, the shape fidelity of the star constructs was quantified using digital image analysis. Immediately after printing, top-view photographs of the constructs next to a metric scale were taken for accurate spatial calibration. These images were then processed using ImageJ software (version 1.53m) (National Institutes of Health, Bethesda, MD, USA). The image scale was calibrated to convert pixel measurements into millimeters. The tip-to-tip length and projected surface area of each construct were then measured and compared with the corresponding theoretical dimensions of the CAD model. Shape fidelity was calculated based on length $\left({SF}_{length}\right)$ and projected area (${SF}_{area}$) using Equations (4) and (5), respectively:

(4) $${SF}_{length}\left(\%\right)=\frac{L_{p}}{L_{t}}\times 100$$

(5) $${SF}_{area}\left(\%\right)=\frac{A_{p}}{A_{t}}\times 100,$$

where $L_{p}$ and $A_{p}$ represent the experimentally measured length and projected area, respectively, and $L_{t}$ and $A_{t}$ represent the corresponding theoretical values obtained from the CAD model. Values closer to 100% indicate closer agreement between the printed construct and the target dimensions. Values above 100% indicate that the measured dimension exceeds the target value, whereas values below 100% indicate that it is smaller.

### 2.8. Evaluation of Weight Loss, Apparent Swelling, and Structural Integrity During Immersion

The weight loss of the 3D printed constructs was determined by measuring their weights before and after the drying process. Immediately after fabrication, the initial weight of the freshly printed star construct ($W_{i}$) was recorded. The samples were then dried in an oven (Faithful, Cangzhou, China) at 45 °C and weighed at 40 and 60 h. Since no significant weight change was observed between these time points, the weight at 60 h was recorded as the final dry weight $W_{0}$. The weight loss percentage was calculated using Equation (6):

(6) $$Weight\;Loss\left(\%\right)=\frac{W_{i}-W_{0}}{W_{i}}\times 100,$$

where $W_{i}$ is the initial weight of the freshly printed constructs (g), and $W_{0}$ is the weight of the fully dried printed constructs (g).

After drying, the constructs were immersed in phosphate-buffered saline (PBS, pH 7.4) (Sigma-Aldrich, St. Louis, MO, USA) at room temperature (~25 °C) to evaluate their structural stability under aqueous conditions. The apparent swelling behavior was evaluated by measuring the wet weight of the constructs after 1 and 2 h of immersion. At each time point, the construct was carefully removed from PBS, excess surface liquid was removed, and the remaining main structure was weighed. After each measurement, the constructs were returned to the same PBS solution for continued immersion. The apparent swelling ratio was calculated using Equation (7):

(7) $$Apparent\;Swelling\;Ratio\left(\%\right)=\frac{W_{t}-W_{0}}{W_{0}}\times 100,$$

where $W_{0}$ is the initial dry weight of the construct (g), and $W_{t}$ is the wet weight of the remaining main structure at the corresponding immersion time (g). Small detached particles were observed during immersion and were not included in the weight measurements. Therefore, the calculated values were expressed as apparent swelling ratios, reflecting the combined effects of liquid uptake and material loss rather than the total water uptake of the original constructs. Quantitative swelling measurements were limited to the first 2 h, as progressive fragmentation at later time points reduced the reliability of gravimetric measurements. The shape retention and physical disintegration of the star constructs were visually evaluated and photographed over a 5-h period.

### 2.9. Statistical Analysis

All quantitative measurements were performed using three independently prepared batches for each formulation (*n* = 3), and the results are presented as mean ± standard deviation (SD). Statistical analysis of the data was conducted using one-way analysis of variance (ANOVA) followed by Tukey’s post hoc test using Python (version 3.13.15) (Python Software Foundation, Wilmington, DE, USA) with the SciPy and statsmodels libraries. Differences were considered statistically significant at *p* < 0.05. In the tables, different lowercase superscript letters (a, b, c) indicate statistically significant differences among formulations.

## 3. Results and Discussion

### 3.1. FTIR Characterization and pH Analysis of the Formulations

**Figure 2:**
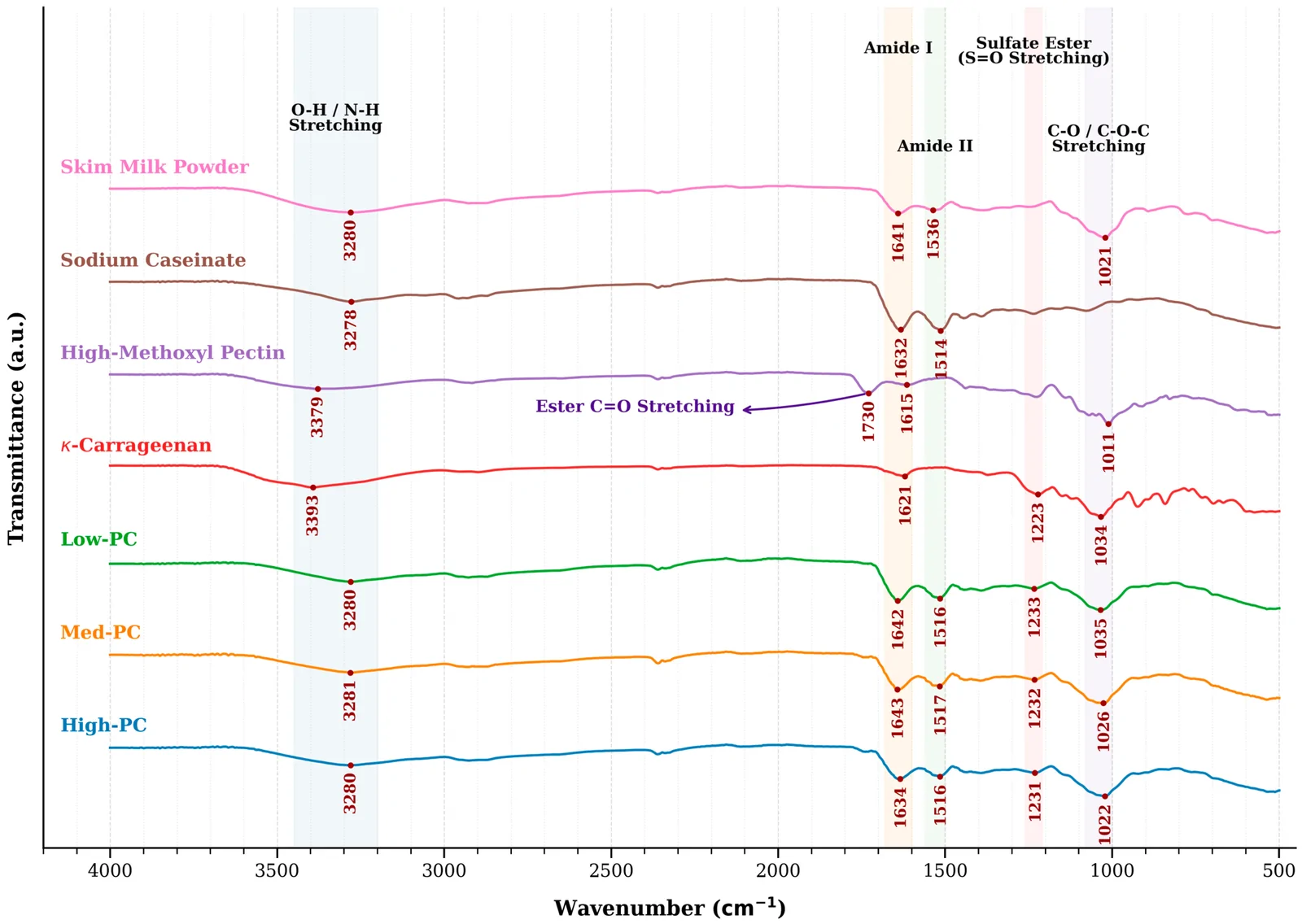
Fourier-transform infrared (FTIR) spectra of the individual biopolymer ingredients (skim milk powder, sodium caseinate, pectin, and κ-carrageenan) and the composite 3D printing food inks (Low-PC, Med-PC, and High-PC). The colored shaded regions highlight selected characteristic absorption bands associated with the functional groups of the components.

The chemical structure and characteristic functional groups of the Low-PC, Med-PC, and High-PC formulations were investigated using FTIR spectroscopy (Figure 2). The spectra of skim milk powder, sodium caseinate, high-methoxyl pectin, and κ-carrageenan were first examined individually to identify the characteristic absorption bands of the components. In the spectra of skim milk powder and sodium caseinate, the Amide I bands were observed at approximately 1641 and 1632 cm^−1^, respectively, while the corresponding Amide II bands appeared at 1536 and 1514 cm^−1^, respectively [26]. The polysaccharide components exhibited characteristic absorption bands associated with C-O and C-O-C stretching vibrations in the 1000–1100 cm^−1^ region [35,36,37]. In addition, high-methoxyl pectin showed an ester carbonyl (C=O) absorption band at approximately 1730 cm^−1^, whereas κ-carrageenan exhibited a characteristic sulfate ester (S=O stretching) band at approximately 1223 cm^−1^ [38].

The spectra of Low-PC, Med-PC, and High-PC retained characteristic absorption bands associated with both protein and polysaccharide components, consistent with their presence in the composite formulations [39]. The Amide I bands were observed at approximately 1642, 1643, and 1634 cm^−1^ for Low-PC, Med-PC, and High-PC, respectively, while the corresponding Amide II bands appeared at 1516, 1517, and 1516 cm^−1^, respectively. These differences in band positions may reflect changes in the local vibrational environment of the protein-containing matrix. However, because the formulations contain multiple components with overlapping absorption bands, the observed spectral differences cannot be attributed exclusively to specific protein–polysaccharide interactions.

A broad absorption band associated with O-H and N-H stretching vibrations was observed in all formulations within the 3200–3500 cm^−1^ region [40,41,42]. The corresponding bands appeared at approximately 3280, 3281, and 3280 cm^−1^ for Low-PC, Med-PC, and High-PC, respectively. Differences in band profiles and intensities relative to the individual components may be associated with variations in hydrogen-bonding environments, hydration, and molecular organization. Nevertheless, these spectral features alone do not establish the formation of specific intermolecular bonds.

The pH values of the formulations were measured at three stages of ink preparation: after preparation, after removal from the refrigerator, and after resting at room temperature. Across all measurement stages, Low-PC exhibited the highest pH, whereas High-PC showed the lowest values. Following the resting period at room temperature, the pH values were 6.613 ± 0.025, 6.457 ± 0.021, and 6.317 ± 0.040 for Low-PC, Med-PC, and High-PC, respectively. These values were above the approximate isoelectric point of casein (pH 4.6), indicating that casein would be expected to carry a net negative charge under the experimental conditions [43]. Since pectin and κ-carrageenan also contain negatively charged groups at these pH values, strong electrostatic complexation between oppositely charged biopolymers is unlikely to be the dominant mechanism governing the formulation structure [21,44]. Instead, hydrogen bonding, competitive hydration, polymer-chain organization, and possible thermodynamic incompatibility may contribute to the observed differences in material behavior. However, the relative contributions of these mechanisms cannot be established from FTIR measurements alone.

No distinct new absorption bands attributable to newly formed covalent functional groups were identified in the composite formulation spectra. Although this observation is consistent with physical mixing and non-covalent interactions, it does not independently exclude all chemical modifications. Overall, the FTIR results demonstrate the presence of characteristic protein and polysaccharide functional groups in the composite formulations, while the pH measurements provide additional context for interpreting their possible interactions. To further investigate the structural characteristics of the formulations, their microstructure was examined using SEM.

### 3.2. Microstructural Morphology of the Formulations

**Figure 3:**
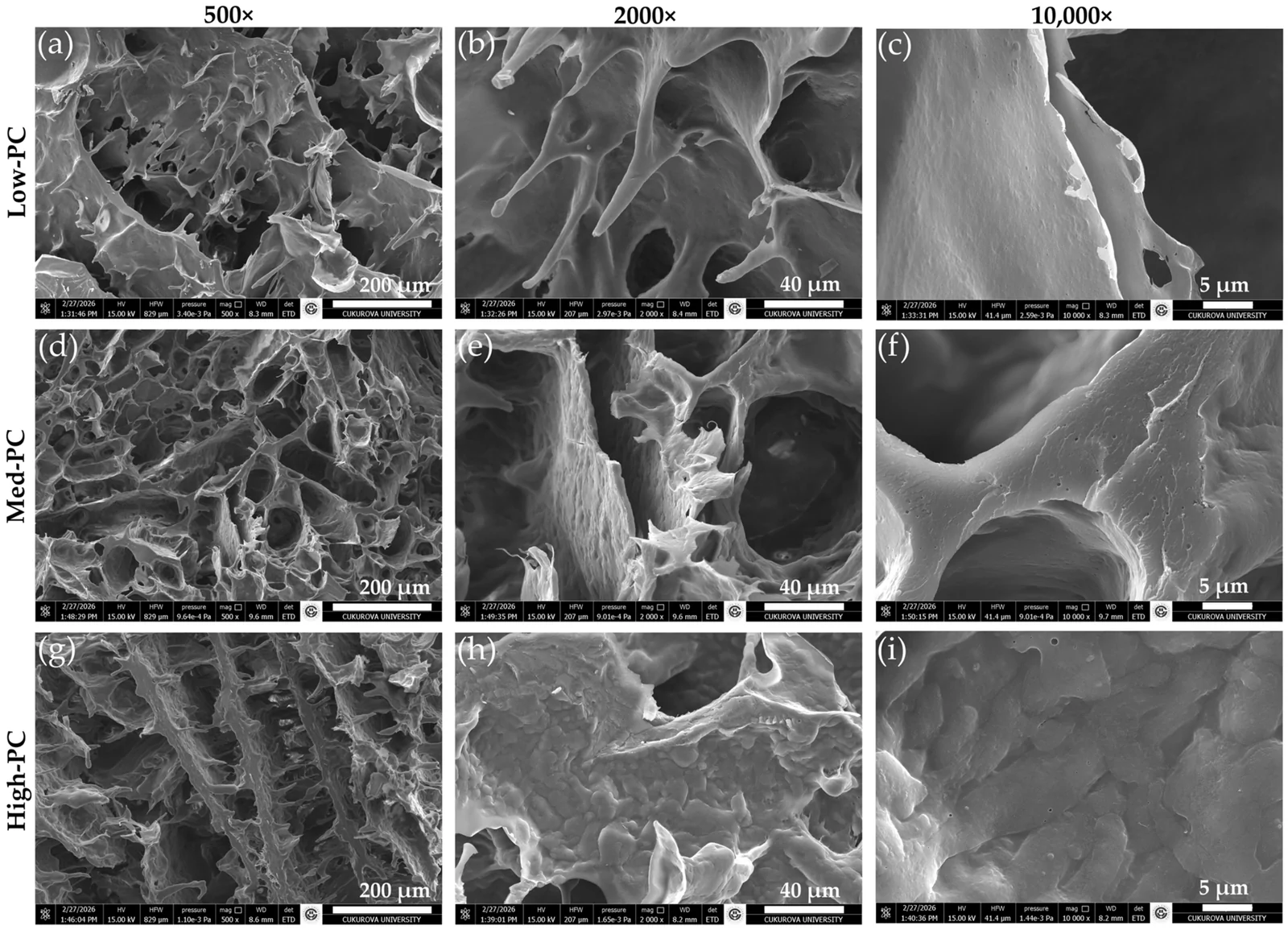
Scanning electron microscopy (SEM) images of the freeze-dried inks at 500× (**left column**), 2000× (**middle column**), and 10,000× (**right column**) magnifications: (**a**–**c**) Low-PC, (**d**–**f**) Med-PC, and (**g**–**i**) High-PC formulations. Scale bars represent 200 µm (**left column**), 40 µm (**middle column**), and 5 µm (**right column**).

The cross-sectional microarchitecture of the lyophilized formulations was imaged using SEM (Figure 3). The micrographs showed clear differences in the morphology of the freeze-dried matrices with increasing PC concentrations. Low-PC had a loose and highly porous internal structure with large visible voids and relatively thin, irregular walls (Figure 3a,b). As the PC concentration increased, the observed matrix morphology generally became more compact. Compared to Low-PC, Med-PC showed a more closely arranged porous structure, with numerous visible voids and interconnected wall-like features (Figure 3d,e). Finally, although some voids were still visible, High-PC exhibited comparatively compact regions and broader solid-looking walls within its porous structure (Figure 3g,h). Higher-magnification SEM images (10,000×) provided additional information on the local surface morphology of the lyophilized formulations (Figure 3c,f,i). Low-PC exhibited a relatively smooth wall surface with fine surface irregularities (Figure 3c). Med-PC exhibited a more irregular surface morphology, with fine surface features and small pit-like structures (Figure 3f). In contrast, High-PC displayed broad, compact-looking regions with locally layered and irregular surface features (Figure 3i). Although these morphological differences were visible, distinct protein-rich clusters and polysaccharide-rich domains could not be conclusively identified from the SEM images alone.

The differences in the observed microstructure may be associated with changes in polymer concentration, molecular organization, and interactions within the protein–polysaccharide matrix [45]. However, SEM images alone cannot confirm polymer-chain entanglement or phase separation. Furthermore, because the samples were examined after lyophilization, freezing and water removal may have influenced the observed pore morphology [46]. Therefore, the images provide qualitative information about the freeze-dried matrices rather than a direct representation of the network organization in the hydrated inks.

A dense internal network is important for mechanical stability in extrusion-based 3D food printing. A more compact network may improve the self-supporting capacity of the printed material and help maintain its shape during deposition. This increasingly compact morphology is consistent with the higher consistency index, yield stress, and storage modulus, and the textural hardness measured at higher PC concentrations. High-PC also exhibited the highest structural recovery, further indicating its ability to recover its structure following deformation. The increases in these metrics indicate the development of a robust composite matrix. The compact structure of High-PC may also be associated with its greater structural integrity during subsequent immersion in PBS. In contrast, the relatively porous and loose network of Low-PC is consistent with its greater tendency toward post-extrusion spreading. The morphological changes from a loose to a tightly packed network are consistent with the variations in rheological and mechanical properties, which are critical for the final printability of the formulations.

### 3.3. Rheological Properties of the Formulated Inks

#### 3.3.1. Steady Shear Flow and Shear-Thinning Behavior

**Figure 4:**
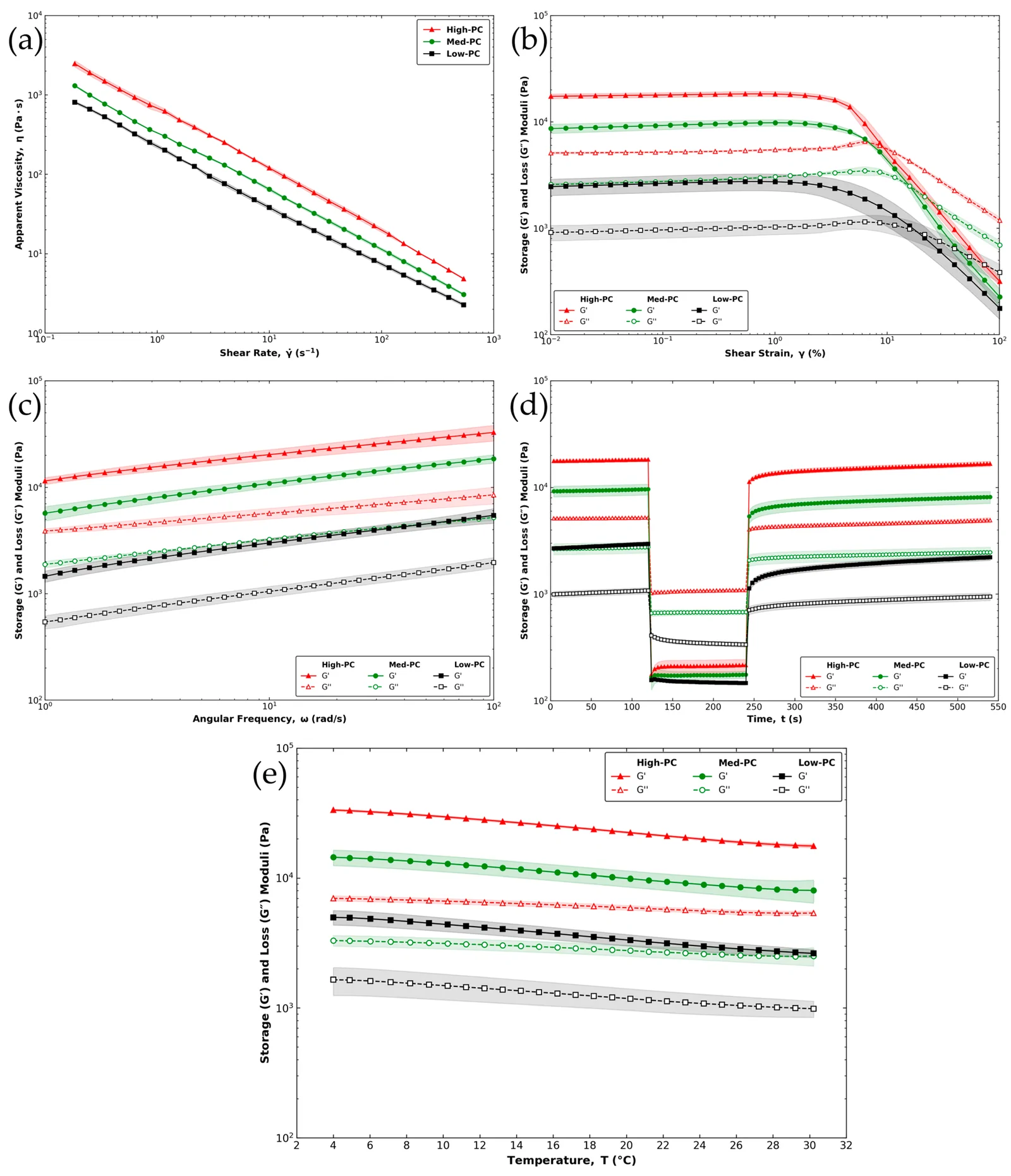
Rheological characterization of Low-PC, Med-PC, and High-PC formulations: (**a**) steady shear flow curves, (**b**) amplitude sweep tests, (**c**) frequency sweep tests, (**d**) three-interval thixotropy (3ITT) tests, and (**e**) temperature sweep tests. The shaded regions indicate the standard deviation (SD, *n* = 3).

The steady shear flow properties play an important role in the extrudability of the inks through the nozzle during the 3D printing process. The flow curves (Figure 4a) showed that all formulations exhibited shear-thinning (pseudoplastic) behavior, where apparent viscosity decreased with increasing shear rate. This behavior was further quantified by fitting the shear stress–shear rate data to the Power-Law model, with the resulting parameters presented in Table 2. The flow behavior index (*n*) values for all inks were below 1, ranging from 0.210 to 0.281, indicating their pseudoplastic behavior. The *n* values decreased significantly with increasing PC concentration (*p* < 0.05), suggesting more pronounced shear-thinning behavior at higher PC concentrations. For extrusion-based 3D printing, this shear-thinning behavior is desirable since it leads to a sharp reduction in viscosity under high shear rates at the nozzle wall, facilitating material flow during extrusion [47]. After extrusion, the removal of shear stress may allow the material to rebuild its internal structure and regain sufficient strength to maintain structural integrity [2].

The consistency index (*K*) also differed significantly among the formulations (*p* < 0.05). Low-PC exhibited a relatively low *K* value of 205.99 $Pa\cdot s^{n}$, suggesting a lower flow resistance and easier flow through the nozzle. Conversely, High-PC exhibited a much higher *K* value of 724.57 $Pa\cdot s^{n}$, approximately 3.5-fold higher than that of Low-PC, indicating greater resistance to flow. For Med-PC, the *K* value was 360.95 $Pa\cdot s^{n}$, indicating a consistency level between that of Low-PC and High-PC. The lower *K* value of Low-PC may make extrusion easier but could reduce its ability to maintain the printed shape after deposition. In contrast, the higher *K* value of High-PC may provide better structural stability but make extrusion more difficult. The intermediate *K* value of Med-PC may have contributed to its balance between extrudability and shape retention, consistent with its printing performance.

Since all formulations exhibited measurable yield stress during amplitude sweep tests, the Herschel–Bulkley model, which incorporates a yield stress parameter, was also evaluated. For Low-PC, the Herschel–Bulkley model provided a higher coefficient of determination ($R^{2}=0.9997)$ than the Power-Law model ($R^{2}=0.9970),$ with fitted parameters of $\tau_{0}$ = 78.43 ± 7.51 Pa, *K* = 141.98 ± 14.64 $Pa\cdot s^{n}$, and *n* = 0.333 ± 0.010. However, for Med-PC and High-PC, the fitted yield stress parameter $(\tau_{0}$) reached the lower bound of zero, effectively reducing the Herschel–Bulkley model to the Power-Law model. Therefore, the Herschel–Bulkley model did not improve the fitting performance for these two formulations. Although Med-PC and High-PC exhibited measurable yield stress in the amplitude sweep tests, the Herschel–Bulkley model did not identify a positive yield stress parameter $\tau_{0}$ from the steady shear data. This difference may be related to distinct approaches used to assess yielding behavior and does not necessarily indicate the absence of a physical yield stress.

#### 3.3.2. Viscoelastic Characteristics and Yield Stress

The structural breakdown and yielding properties were analyzed using amplitude sweep tests. As shown in Figure 4b, *G*′ was much higher than *G*″ across the LVR, indicating solid-like behavior for all inks. The internal structures of the inks remained stable within their LVRs. Beyond these regions, *G*′ began to decrease, indicating the progressive breakdown of the internal network. Med-PC exhibited the highest mean LVR of 2.42%, while Low-PC and High-PC showed 2.05% and 1.63%, respectively (Table 3). However, these differences were not statistically significant (*p* > 0.05). This suggested that the formulations exhibited comparable limits of linear viscoelastic behavior under the tested conditions. On the other hand, the yield stress increased significantly with PC concentration, with values of 74.40 Pa, 338.10 Pa, and 554.00 Pa for Low-PC, Med-PC, and High-PC, respectively (*p* < 0.05). The much higher yield stress in High-PC indicated a much stronger internal network [48]. Although it may help the ink retain its shape after deposition, it may also increase the required extrusion pressure [49]. The flow stress (*G*′ = *G*″) values showed a similar trend, increasing from 240.43 Pa for Low-PC to 562.53 Pa for Med-PC, and up to 758.03 Pa for High-PC, with significant differences among all formulations (*p* < 0.05). This showed that High-PC required a much greater stress to reach a predominantly viscous response under oscillatory deformation. Med-PC, however, showed a favorable balance for 3D printing. Its moderate yield and flow stresses may allow the ink to be extruded under applied pressure while still providing enough structural strength to hold its shape after deposition [50].

The frequency-dependent viscoelastic behavior of the inks was further evaluated using frequency sweep tests. As shown in Figure 4c, *G*′ was consistently greater than *G*″ across the entire frequency range for all formulations. This indicated that the elastic-dominant behavior was maintained across the tested frequency range. The inks exhibited frequency-dependent viscoelastic behavior since both *G*′ and *G*″ increased with increasing angular frequency. At 10 rad/s, the *G*′ values of Low-PC, Med-PC, and High-PC were 2990 Pa, 10,854 Pa, and 20,251 Pa, respectively (Table 3). High-PC showed a notably higher *G*′ than the other formulations, indicating a far stronger elastic network (*p* < 0.05). This finding is consistent with its high yield and flow stresses observed in the amplitude sweep. On the other hand, Low-PC had the lowest *G*′, pointing to a relatively weaker elastic structure. Med-PC showed an intermediate *G*′, indicating an elastic network that was stronger than that of Low-PC but weaker than that of High-PC. The loss factor (*tan δ*) values at 10 rad/s were 0.350, 0.299, and 0.281 for Low-PC, Med-PC, and High-PC, respectively. Because *tan δ* remained below 1 in all formulations, their elastic response dominated over their viscous response. Low-PC had the highest *tan δ* value, which was significantly greater than that of Med-PC and High-PC (*p* < 0.05), indicating a relatively greater viscous contribution. However, no significant difference was observed between Med-PC and High-PC (*p* > 0.05). The intermediate elastic strength of Med-PC may have contributed to its ability to support deposited layers, although its printing performance cannot be explained by oscillatory properties alone [10].

#### 3.3.3. Structural Recovery (3ITT Analysis)

The structural recovery behavior of the inks was evaluated using a 3ITT. This test provides a rheological representation of the structural changes occurring during extrusion-based 3D printing (Figure 4d). The initial low-strain interval represents the initial structural state of the ink. The high-strain interval mimics structural disruption experienced during extrusion. The final low-strain interval represents the post-extrusion recovery phase after deposition. In this phase, the internal network is expected to recover enough strength to retain its shape. As shown in Figure 4d, *G*′ decreased for all formulations during the high-strain interval. When the strain was reduced, *G*′ increased again, indicating partial recovery of the internal network.

At 60 s, High-PC showed the highest mean recovery of 77.36%, followed by Med-PC (71.71%) and Low-PC (56.69%) (Table 3). Both High-PC and Med-PC exhibited significantly higher recovery than Low-PC (*p* < 0.05), whereas the difference between High-PC and Med-PC was not statistically significant (*p* > 0.05). This indicated that High-PC and Med-PC showed greater short-term structural recovery than Low-PC after deformation. This recovery may contribute to the ability of the deposited filament to resist deformation and retain its printed shape.

At 300 s, the recovery values increased to 74.81%, 84.43%, and 91.43% for Low-PC, Med-PC, and High-PC, respectively. High-PC and Med-PC again exhibited significantly higher recovery than Low-PC (*p* < 0.05), without a significant difference between them (*p* > 0.05). Although High-PC showed the highest mean structural recovery, Med-PC achieved the closest agreement with the target geometry during printing. This suggests that higher recovery does not necessarily correspond to higher printing accuracy. Overall, the steady-shear and viscoelastic properties, together with the 3ITT results, suggest that Med-PC provided intermediate flow resistance and elastic strength, whereas High-PC exhibited greater rheological strength and the highest mean structural recovery. These differences highlight the importance of balancing rheological properties to achieve the desired printing performance.

#### 3.3.4. Temperature-Dependent Viscoelastic Behavior

The temperature-dependent viscoelastic behavior of the inks was evaluated using temperature sweep tests (Figure 4e). As the temperature increased from 4 to 30 °C, *G*′ remained higher than *G*″ for all formulations, indicating that elastic-dominant behavior was maintained throughout the investigated temperature range. Both moduli generally decreased with increasing temperature, suggesting a gradual reduction in viscoelastic strength during heating. High-PC exhibited the highest *G*′ values, followed by Med-PC and Low-PC, consistent with the results of the frequency sweep tests. No crossover between *G*′ and *G*″ was observed, indicating that no distinct transition from elastic-dominant to viscous-dominant behavior occurred within the tested temperature range. These results suggest that the formulations maintained their predominantly elastic characteristics during heating, although their viscoelastic strength decreased with increasing temperature.

### 3.4. Mechanical and Textural Properties

The mechanical and textural properties of the inks were investigated using TPA, and the results are detailed in Table 4. The hardness of the inks increased with increasing PC concentration, from 0.68 N for Low-PC to 1.45 N for Med-PC and 3.14 N for High-PC. Gumminess significantly increased from 0.33 N for Low-PC to 0.67 N for Med-PC and 1.23 N for High-PC. For chewiness, High-PC (0.99 N) was significantly higher than both Med-PC (0.53 N) and Low-PC (0.30 N), which showed no statistical difference from each other. The springiness (0.78–0.92), cohesiveness (0.39–0.48) and resilience (0.09–0.11) of the inks did not significantly change with increasing PC concentration.

The enhancement in hardness, gumminess, and chewiness at higher PC concentrations is in strong agreement with the increased rheological values (consistency index, yield stress, and storage modulus), supporting the development of a stronger and more rigid structure. This increase in mechanical strength was also consistent with the denser microstructure observed in the SEM images. Increasing the PC concentration mainly enhanced the firmness-related properties of inks, without significantly changing their springiness, cohesiveness, and resilience. The TPA results also indicated that increasing PC concentration progressively reinforced the composite ink structure, while Med-PC exhibited intermediate textural properties between Low-PC and High-PC.

From a sensory perspective, the higher hardness and chewiness of High-PC may result in a firmer texture and greater resistance during chewing, whereas Med-PC may provide a comparatively softer texture. These differences could influence mouthfeel and consumer acceptance, although sensory evaluation would be required to confirm their effects.

However, since TPA was performed on molded samples, these results may not fully reflect the mechanical properties of 3D-printed structures, which also depend on interlayer bonding.

### 3.5. Six-Axis Robotic 3D Printing Performance, Shape Fidelity, and Post-Print Stability

**Figure 5:**
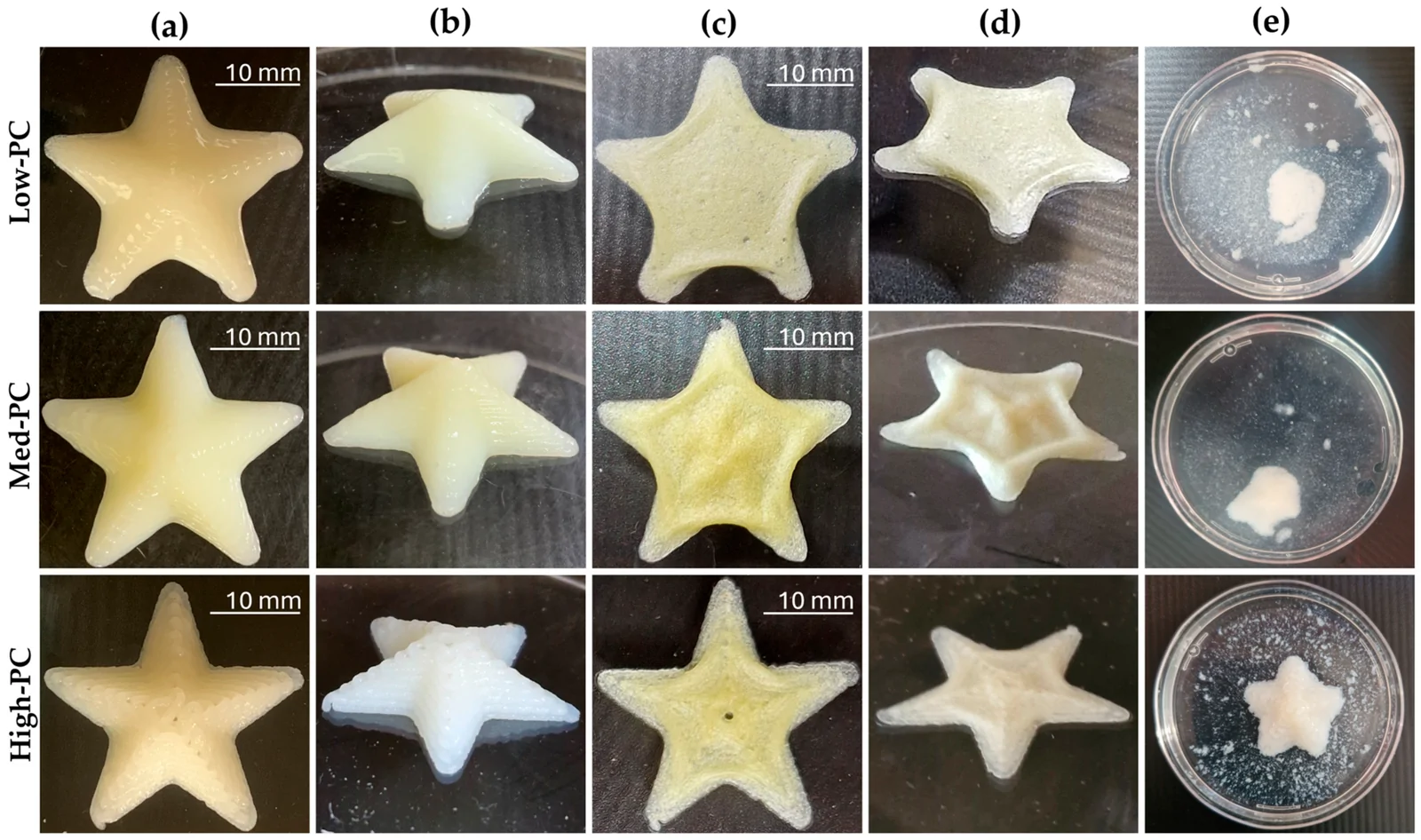
Six-axis robotic 3D printed star-shaped structures of Low-PC, Med-PC, and High-PC inks at different post-printing stages: (**a**) top-view images immediately after printing, (**b**) isometric-view images immediately after printing, (**c**) top-view images of the dried constructs, (**d**) isometric-view images of the dried constructs, and (**e**) constructs after 5 h of immersion. Scale bars represent 10 mm.

The printing and shape fidelity of the inks were evaluated by printing a five-pointed star geometry converging to a central apex. As shown in Figure 5, although differences were observed in the extrusion behavior and the preservation of the printed geometries, all inks were successfully extruded through the nozzle and deposited to form the targeted structure. Macroscopic evaluation of the printed constructs revealed distinct variations in strand fusion quality. The low yield stress of Low-PC was associated with excessive material spreading, resulting in poorly defined individual printing lines. In contrast, High-PC produced more clearly distinguishable filaments, with less apparent fusion between adjacent strands. Med-PC, however, exhibited well-defined printing lines with more uniform strand fusion. These macroscopic differences were quantified by comparing the printed structures to the CAD model, and the calculated area and length fidelity values are presented in Table 5.

The results demonstrate that PC concentration significantly affected both length- and area-based shape fidelity. For area-based shape fidelity (${SF}_{area}$), Low-PC, Med-PC, and High-PC exhibited values of 109.73%, 96.16%, and 88.93%, respectively, with statistically significant differences among all inks (*p* < 0.05). Relative to the target area (100%), Low-PC produced an oversized structure, consistent with greater material spreading and lower structural rigidity. In contrast, High-PC, with an ${SF}_{area}$ value of 88.93%, generated an area smaller than the target geometry, which may be related to its high yield stress and stronger internal network [51]. Among the inks, Med-PC showed the closest agreement with the target area, with an ${SF}_{area}$ of 96.16%.

The length-based shape fidelity (${SF}_{length}$) values were 97.21%, 94.42%, and 91.53% for Low-PC, Med-PC, and High-PC, respectively, with significant differences among all inks (*p* < 0.05). Although Low-PC showed the highest length fidelity (97.21%), this single-dimensional parameter does not capture lateral spreading. The relatively low yield stress of Low-PC may have allowed greater lateral deformation, contributing to its enlarged printed area. Med-PC showed a lower length fidelity than Low-PC but provided the closest agreement with the target area. Its intermediate consistency and yield stress therefore appeared to provide a favorable balance between extrusion flowability and post-deposition structural stability. Although High-PC exhibited the highest mean structural recovery in the 3ITT measurements, this did not result in the highest shape fidelity under the tested printing conditions. This indicates that structural recovery alone does not determine printing accuracy, which also depends on extrusion behavior and material deposition.

The post-printing drying behavior of the printed constructs was evaluated based on weight loss measurements, and the results are presented in Table 5. The highest weight loss was observed for the Low-PC construct at 80.83%. With increasing PC concentration, the weight loss significantly decreased to 78.50% for Med-PC (*p* < 0.05). However, no statistically significant difference was found between High-PC (77.48%) and Med-PC. These results indicate that increasing PC concentration from Low-PC to Med-PC was associated with a significant reduction in weight loss, whereas the further increase from Med-PC to High-PC did not result in a statistically significant additional reduction.

The structural stability of the dried star-shaped constructs in an aqueous medium was evaluated through visual assessment of structural integrity and apparent swelling measurements during immersion in PBS. After 1 h of immersion, the swelling ratios were 252.00%, 265.08%, and 241.44% for Low-PC, Med-PC, and High-PC, respectively. After 2 h, these values increased to 267.81%, 313.81%, and 285.12%, respectively (Table 5). No statistically significant differences in swelling ratio were observed among the formulations at either time point (*p* > 0.05). However, partial disintegration during immersion may have affected the measured swelling ratios due to material loss, potentially leading to an underestimation of water uptake. Therefore, structural integrity after 5 h was assessed qualitatively by visually examining how well the constructs retained their printed geometry. As shown in Figure 5, distinct differences in structural stability were observed among the constructs after the 5 h immersion period. Low-PC and Med-PC lost their original star-shaped geometry and showed extensive macroscopic disintegration. In contrast, High-PC retained a recognizable star-like central structure at the end of the observation period, although some disintegrated material was also observed around the remaining structure. This observation suggests that the internal network of High-PC may have provided greater resistance to structural disruption during immersion. The greater structural integrity of High-PC was consistent with its denser microstructure observed by SEM and its rheological and textural properties, including higher yield stress, storage modulus, structural recovery, and hardness. However, the visual observations after 5 h should not be interpreted as evidence of statistically significant differences in disintegration or swelling behavior.

Overall, the printing and post-printing results showed that PC concentration affected different aspects of structural performance. Med-PC provided the closest agreement with the target area, whereas Low-PC showed the highest length fidelity. High-PC, despite its lower dimensional fidelity, exhibited the highest mean structural recovery and visually retained greater structural integrity during aqueous immersion. Although the swelling ratios did not differ significantly among the formulations during the first 2 h, the qualitative observations after 5 h suggested differences in the preservation of the printed geometry. These findings highlight the importance of balancing flowability and structural strength to obtain printable food inks with adequate post-printing stability.

## 4. Conclusions

This study demonstrated that the polysaccharide (pectin and κ-carrageenan) concentration in formulations with a skim milk and sodium caseinate matrix had distinct effects on their molecular, microstructural, rheological, mechanical, and printing properties. FTIR results suggested possible non-covalent interactions between the formulation components, although their specific nature could not be confirmed. SEM images showed that the freeze-dried matrix became gradually more compact as the PC concentration increased. All formulations exhibited shear-thinning behavior, while increasing PC concentration generally increased rheological strength and textural hardness.

In the 3ITT test, High-PC showed the highest mean structural recovery at both 60 and 300 s, although its recovery did not differ significantly from Med-PC (*p* > 0.05). During six-axis robotic printing, the Low-PC formulation spread beyond the target geometry, while High-PC produced smaller structures with distinct printing lines. Med-PC, however, closely matched the target geometry, showing an area-based shape fidelity of 96.16%. Thus, the formulation with the highest structural recovery did not provide the highest printing accuracy under the tested conditions.

After 2 h of immersion, the swelling ratios did not differ significantly among the formulations (*p* > 0.05). However, visual observations after 5 h showed that the High-PC formulation retained a recognizable star-like structure, while Low-PC and Med-PC lost their original printed geometries in the aqueous environment. Overall, Med-PC provided the closest agreement with the target printed area, whereas High-PC showed the highest mean structural recovery and better visual preservation during immersion. These findings highlight the need to balance printing accuracy and post-printing structural stability when developing food inks for six-axis robotic 3D printing.

## Figures and Tables

**Table 1:** Composition of the ink formulations (expressed as weight percentages, wt%).

Ink	Sodium Caseinate	Polysaccharide Complex	Skim Milk Powder	Distilled Water
Low-PC	8.00	2.00	9.000	81.000
Med-PC	8.00	3.25	8.875	79.875
High-PC	8.00	4.50	8.750	78.750

**Table 2:** Power-Law model parameters obtained from the shear stress–shear rate data of Low-PC, Med-PC, and High-PC formulations.

Parameters	Low-PC	Med-PC	High-PC
$\mathrm{Consistency}\;\mathrm{Index},\;K\;(Pa\cdot s^{n})$	205.99 ± 10.45 ^c^	360.95 ± 8.74 ^b^	724.57 ± 42.49 ^a^
Flow Behavior Index, *n*	0.281 ± 0.002 ^a^	0.245 ± 0.003 ^b^	0.210 ± 0.009 ^c^
$R^{2}$	0.9970 ± 0.0011 ^a^	0.9982 ± 0.0008 ^a^	0.9898 ± 0.0117 ^a^

Values are expressed as mean ± standard deviation (*n* = 3). Different superscript letters (a–c) in the same row indicate statistically significant differences (*p* < 0.05).

**Table 3:** Rheological parameters of the formulated inks.

Parameters	Low-PC	Med-PC	High-PC
LVR ($\gamma_{LVR}$, %)	2.05 ± 0.38 ^a^	2.42 ± 0.44 ^a^	1.63 ± 0.09 ^a^
Yield Stress ($\tau_{y},\;Pa)$	74.40 ± 28.56 ^c^	338.10 ± 14.99 ^b^	554.00 ± 44.26 ^a^
Flow Stress ($\tau_{f},\;Pa)$	240.43 ± 71.87 ^c^	562.53 ± 34.03 ^b^	758.03 ± 82.95 ^a^
*G*′ at 10 rad/s (Pa)	2990 ± 312 ^c^	10854 ± 996 ^b^	20251 ± 2271 ^a^
*tan δ* at 10 rad/s	0.350 ± 0.004 ^a^	0.299 ± 0.027 ^b^	0.281 ± 0.002 ^b^
Recovery at 60 s (%)	56.69 ± 2.88 ^b^	71.71 ± 2.85 ^a^	77.36 ± 2.90 ^a^
Recovery at 300 s (%)	74.81 ± 3.02 ^b^	84.43 ± 3.06 ^a^	91.43 ± 4.71 ^a^

Values are expressed as mean ± standard deviation (*n* = 3). Different superscript letters (a–c) in the same row indicate statistically significant differences (*p* < 0.05).

**Table 4:** Texture Profile Analysis (TPA) parameters of the formulated inks.

Ink	Hardness (N)	Springiness	Cohesiveness	Gumminess (N)	Chewiness (N)	Resilience
Low-PC	0.68 ± 0.28 ^c^	0.92 ± 0.02 ^a^	0.48 ± 0.03 ^a^	0.33 ± 0.12 ^c^	0.30 ± 0.12 ^b^	0.09 ± 0.02 ^a^
Med-PC	1.45 ± 0.16 ^b^	0.78 ± 0.11 ^a^	0.47 ± 0.06 ^a^	0.67 ± 0.03 ^b^	0.53 ± 0.10 ^b^	0.10 ± 0.01 ^a^
High-PC	3.14 ± 0.28 ^a^	0.80 ± 0.12 ^a^	0.39 ± 0.03 ^a^	1.23 ± 0.04 ^a^	0.99 ± 0.17 ^a^	0.11 ± 0.01 ^a^

Values are expressed as mean ± standard deviation (*n* = 3). Different superscript letters (a–c) in the same column indicate statistically significant differences (*p* < 0.05).

**Table 5:** Quantitative evaluation of shape fidelity (area and length) of freshly printed geometries, their weight loss after drying, and apparent swelling ratios after 1 and 2 h of immersion in PBS.

Ink	Shape Fidelity		Weight Loss (%)	Swelling Ratio (%)	
	Area (%)	Length (%)		After 1 h	After 2 h
Low-PC	109.73 ± 0.40 ^a^	97.21 ± 0.94 ^a^	80.83 ± 0.91 ^a^	252.00 ± 43.67 ^a^	267.81 ± 59.30 ^a^
Med-PC	96.16 ± 0.31 ^b^	94.42 ± 0.63 ^b^	78.50 ± 0.39 ^b^	265.08 ± 25.96 ^a^	313.81 ± 15.44 ^a^
High-PC	88.93 ± 0.24 ^c^	91.53 ± 0.72 ^c^	77.48 ± 0.43 ^b^	241.44 ± 54.58 ^a^	285.12 ± 27.52 ^a^

Values are expressed as mean ± standard deviation (*n* = 3). Different superscript letters (a–c) in the same column indicate statistically significant differences (*p* < 0.05).

## Data Availability

The original contributions presented in this study are included in the article. Further inquiries can be directed to the corresponding author.

## Abbreviations

The following abbreviations are used in this manuscript:

PC	Polysaccharide complex
FTIR	Fourier-transform infrared spectroscopy
ATR	Attenuated total reflectance
SEM	Scanning electron microscopy
TPA	Texture profile analysis
LVR	Linear viscoelastic region
3ITT	Three-interval thixotropy test
CAD	Computer-aided design
SF	Shape fidelity
